# Peptidyl Arginine Deiminase Type 4 Gene Promoter Hypo-Methylation in Rheumatoid Arthritis

**DOI:** 10.3390/jcm9072049

**Published:** 2020-06-30

**Authors:** Bogdan Kolarz, Marek Ciesla, Magdalena Dryglewska, Maria Majdan

**Affiliations:** 1 College of Medical Sciences, University of Rzeszow, al. Kopisto 2A/24, 35-359 Rzeszow, Poland; ciesla_marek@wp.pl; 2 Department of Rheumatology and Connective Tissue Disease, Medical University of Lublin, al. Raclawickie 1, 20-059 Lublin, Poland; mdryglewska@wp.pl (M.D.); maria.majdan@gmail.com (M.M.)

**Keywords:** rheumatoid arthritis, DNA methylation, epigenetics, PAD4 gene, RA markers

## Abstract

Protein citrullination is carried out by peptidylarginine deiminase type 4 (PAD4) enzyme. As a consequence of this process, post-translationally modified proteins are formed that become antigens for anti-citrullinated protein antibodies (ACPA). The study aimed at identifying whether the *PADI4* gene is subject to epigenetic regulation through methylation of its promoter region, whether the degree of methylation differs in healthy individuals vs. rheumatoid arthritis (RA) patients and changes in correlation with ACPA, anti-PAD4 and disease activity. A total of 125 RA patients and 30 healthy controls were enrolled. Quantitative real-time methylation-specific PCR was used to analyze the methylation status. ACPA and anti-PAD4 antibodies were determined in serum by enzyme-linked immunosorbent immunoassay. The differences were observed in the degree of *PADI4* gene promoter methylation between RA patients and HC, along with an upward trend for the methylation in RA, which was inversely proportional to the disease activity. A weak or modest negative correlation between the degree of *PADI4* gene methylation and anti-PAD4, disease activity score (DAS28) and ACPA level has been found. The elevated methylation is associated with lower disease activity, lower levels of ACPA and aPAD4. The methylation degree in this area is growing up during effective treatment and might play a role in the RA pathophysiology and therefore could be a future therapeutic target.

## 1. Introduction

Rheumatoid Arthritis (RA) is a chronic, progressive, autoimmune inflammatory disease affecting various organs and tissues, predominantly the synovial membrane, leading to joint destruction [1]. Anti-citrullinated protein antibodies (ACPA) are important markers of RA, recognized as being the most specific. Starting from 2010, along with rheumatoid factor (RF), ACPA are used as serological markers according to the classification criteria of the American College of Rheumatology (ACR) and the European League Against Rheumatism (EULAR) [2]. Their sensitivity and specificity for the diagnosis of RA are 64.9% and 97.9%, respectively [3]. They recognize post-translationally modified auto-antigens generated by the peptidylarginine deiminases (PADs) enzymes family, mostly type 4 (PAD4), which transform arginine to new amino acid citrulline in fibrinogen, α-enolase, vimentin, filaggrin, collagen type I and type II and other various proteins, resulting in the production of immunogenic neoepitopes [4]. There are five isoforms of PADs. PAD4 is found in neutrophils, monocytes, eosinophils, spleen, secretory glands and is connected to myeloid differentiation. The antibodies detected in RA can also be directed against PAD4, but their diagnostic application has not yet been established [5,6,7]. Following the ENSEMBL database the *PADI4* gene is placed on chromosome 1 in location 17,308,195–17,364,004 on the forward strand with references to assembly GRCh38. The gene has 5 transcript variants. However only 2 of them have an open reading frame (ORF) and can form transcripts called *PADI-201* and *PADI4-202*. The first consists of 663 amino acids (aa) and molecular weight 74.1 kDa, the second one 127 aa and 13.1 kDa [8].

The activity of PAD4 demands supraphysiologic calcium concentration, but the presence of anti-PAD4 auto-antibodies (anti-PAD4) may reduce the PADs calcium requirements to the physiological scope [9,10]. The synthesis of anti-PAD4 may facilitate the production of citrullinated proteins and contribute to the formation of ACPA [11]. The post-translational proteins modifications like homocitrullination or citrullination lead to the synthesis of anti-carbamylated protein antibodies (aCarP) or ACPA, respectively. This may play a crucial role in RA pathogenesis. The data indicate that the presence of aCarP and ACPA predates RA development by about 7 (4–10) and 6 years (3–10), respectively. RF can be detected 2 (1–5) years before the onset of the symptoms [1,12]. In other studies, RF was found to be present on average 6 years before disease onset [13]. The citrullination leads to the synthesis of ACPA to a significant extent [14]. This means that citrullination is a very important process causing immunization in RA, and activity of PAD4 enzyme can play a significant role in the pathogenesis of the disease. The chronic presence of post-translationally modified proteins (as a consequence of the infections of *Porphyromonas gingivalis* or *Aggregatibacter actinomycetemcomitans* and the action of other environmental factors, e.g., cigarette smoking) leads to the production of ACPA.

DNA methylation plays a key role in the control of gene expression. The process concerns CpG islands in the promoter regions of about 75% of genes and leads to gene silencing when over-expressed [15]. Epigenetic mechanisms are considered a way of transmitting information from the environment to the inside of a cell through increasing or decreasing gene expression. This process occurs mainly before transcription (DNA methylation, histone modifications), but some of the regulatory mechanisms may also influence the formation of the final gene product after the creation of mRNA (a huge variety of non-coding RNAs) [16,17]. An increasing number of studies are performed to better understand the role DNA methylation in rheumatoid arthritis, from initial studies showing the hypo-methylation of the genome to advanced epigenome studies using modern technology, which can identify differentially methylated genes (DMGs) and discover new candidate genes involved in RA [16,18,19]. No studies on methylation of the *PADI4* gene promoter have been conducted so far.

The expression of the *PADI4* gene may be the *spiritus movens* of all processes leading to the development of RA and include environmental and disease-modifying factors. As the role of ACPA is well documented, it is important to determine whether epigenetic mechanisms, especially DNA methylation, are involved in protein citrullination and indirectly ACPA synthesis. Therefore, the primary aim of our study is to check whether the promoter region of the *PADI4* gene is susceptible to epigenetic regulation by methylation and whether the degree of methylation is connected to DAS28 activity in RA group and compare this to healthy individuals. If such regulation takes place, we assume the lower degree of *PADI4* methylation in RA vs. HC and the trend of decreasing methylation along with increasing disease activity. The relationships between the concentrations of anti-PAD4, ACPA and the disease activity and *PADI4* methylation will also be evaluated.

## 2. Experimental Section

### 2.1. Patients

A total number of 155 unrelated patients, 125 with RA, 82.4% female, aged 52.2 ± 12.3 years (mean ± SD), and 30 healthy controls (HC), 76.7% female, aged 53.2 ± 8.1 years, were enrolled. The characteristics of the subjects are presented in Table 1. With the consent of all those taking part, whole blood and serum samples were collected from patients and stored at −80 °C until analysis. DNA was extracted from whole blood and stored at −80 °C until analysis. RA patients recruited to the study included those consecutively seen at the Department of Rheumatology and Connective Tissue Diseases, Medical University of Lublin, Poland, during March 2016 to April 2017 and can be considered representative of a larger RA population. RA diagnosis was made according to the 2010 ACR/EULAR or 1987 ACR criteria for classification of RA depending on time of diagnosis. Exclusion criteria included the presence of any infection or another severe illness during hospitalization. The healthy controls (HC) were a group of patients with no joint complaint or diagnosed as osteoarthritis, with no inflammatory rheumatic and musculoskeletal diseases. Written informed consent was obtained from every participant before entering the study. The study was conducted in accordance with the Declaration of Helsinki, and the protocol was approved by the Bioethics Board at the Medical University in Lublin, protocol number KE-0254/7/2016. To determine the RA activity, we used the most frequently used DAS28 index. The result >5.1 is described as high disease activity, over 3.2 and up to 5.1 is interpreted as moderate activity, less than 3.2 but 2.6 or more as low disease activity and below 2.6 as remission [20]. DAS28 is the main measurement assessing the degree of disease activity, including remission. The essential information concerning DAS28 and the method of scoring is described in the Supplementary File S2.

### 2.2. DNA Extraction and Methylation

DNA was extracted from 200 µL of frozen whole blood according to the manufacturer’s protocol using the GeneMATRIX Quick Blood DNA Purification Kit (silica spin columns, Eurx, Poland). DNA was eluted in 100 µL and stored at −80 °C until analysis. DNA (1 µg) was converted by sodium bisulphite using the EZ DNA Methylation Gold Kit (Zymo Research, USA) according to the manufacturer’s recommendation, but elution volume was increased to 50 µL (instead of the recommended 10 µL). Quantitative real-time methylation-specific PCR (qMSP) was used to analyze the methylation status.

The *PADI4* promoter was evaluated by the following set of primers: 5′-AGTTTAGGGGTTTTTTATAGTTAGAGGGAC-3′ [sense] and 5′-ATCAAAATACCCAACACACACACG-3′ [antisense]. The promoter region was found in The Eukaryotic Promoter Database. Primers flank the transcription start site (TSS) at positions from −18 to +99 (GRCh38:1:17308178:17308296) [21]. The studied region consists of 5 CpG sites surrounding TSS and in our opinion may have an important effect on gene transcription [22]. The method of choosing CpG sites is shown in Supplementary File S5. The primers were designed in silico by MethPrimer Software, version 1.0 and were complementary to the methylated target sequence. To normalize the input of DNA after bisulfide conversion, the promoter region free of CpG sites in Beta-actin gene (ACTB) was amplified. The ACTB primers were the following sequences: 5′-GGTGGTGATGGAGGAGGTTTAG-3′ [sense] and 5′-CCCTTAAAAATTACAAAAACCACAACC-′3 [antisense]. Primers flanked similar region presented by Menigatti M. et al. [23]. However, their sequences were manually redesigned. The qMSP reaction contained for *PADI4* 150 nM each primer and for ACTB 600 nM as well as 2 µL bisulfide treated DNA.

PCR was performed by Power SybrGreen (Life Technologies) on the COBAZ z480 Real Time PCR System under the following thermal cycling conditions: 95 °C for 10 min—polymerase activation step, followed by 40 cycles: 95 °C for 10 s and annealing/extension step at 63 °C for 1 min, followed by the melting-curve step. The ability of primers to amplify specific sequences was evaluated by using the fully methylated and unmethylated DNA controls (EpiTect PCR Control DNA Set, Qiagen, Germany). QMSP efficiency, both for ACTB and *PADI4* were evaluated based on the Livak and Schmittgen method [24]. The normalized relative ratio based on the Pfaffl method was applied to evaluate a fold-change in the methylation level [25].

### 2.3. Detection of ACPA and Anti-PAD4

ACPA IgG (DiaMetra, Italy) and anti-PAD4 antibodies (IgM, IgG, IgA) (CAYMAN Chemical, Ann Arbor, MI, USA) were determined in serum by enzyme-linked immunosorbent immunoassay (ELISA) and an absorbance reader (Tecan infinite M200 Pro reader and Magellan software, version 7.1). All procedures were prepared according to the manufacturer’s recommendation. The reference range for ACPA had a cut-off of 30 U/mL. The anti-PAD4 reference interval was estimated based on results in the control group as Mean ± 2SD, and a cut-off was 615.24 U/mL. Antibody titers above this range were evaluated as positive.

### 2.4. Statistical Analyses

Depending on the distribution, as assessed by the Shapiro–Wilk *W* test, quantitative values were presented as median (interquartile range), mean ± SD, or numbers with percentages. The relationship between two continuous variables was analyzed by the Spearman’s correlation coefficient. The Whitney–Mann *U* test was used to evaluate differences in methylation level between the two groups (controls vs. RA patients). Kruskal–Wallis ANOVA and multiple comparison analysis post-hoc testing were used to evaluate the differences between control subjects and patients who were divided into groups based on DAS28 scoring. A *p*-value < 0.05 was considered statistically significant. Analysis was performed with STATISTICA Version 13 (StatSoft Inc., Tulsa, OK, USA).

## 3. Results

We found lower PAD4 gene methylation status and higher anti-PAD4 serum level in RA vs. HC. The RA patients were divided according DAS28 scores and results are shown in Table 2.

**Figure 1:**
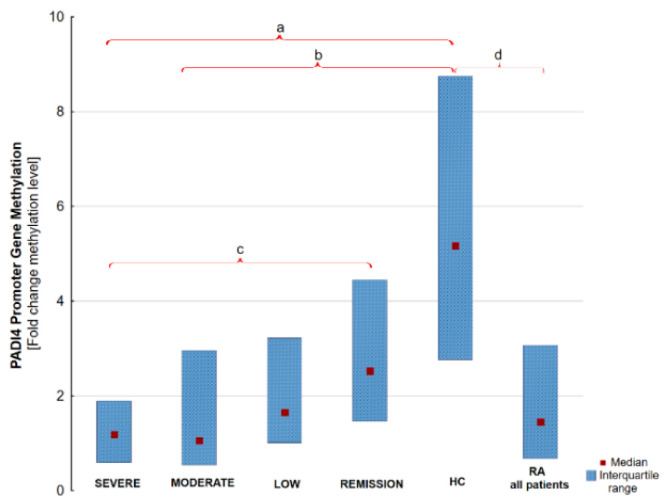
*PADI4* promoter gene methylation level in RA groups according to DAS28 activity, HC and all RA patients. Results presented as median and interquartile range. (a) *p* = 0.00002; (b) *p* = 0.00004; (c) *p* = 0.023; (d) *p* = 0.00001, Remission vs. HC *p* = 0.07.

**Figure 2:**
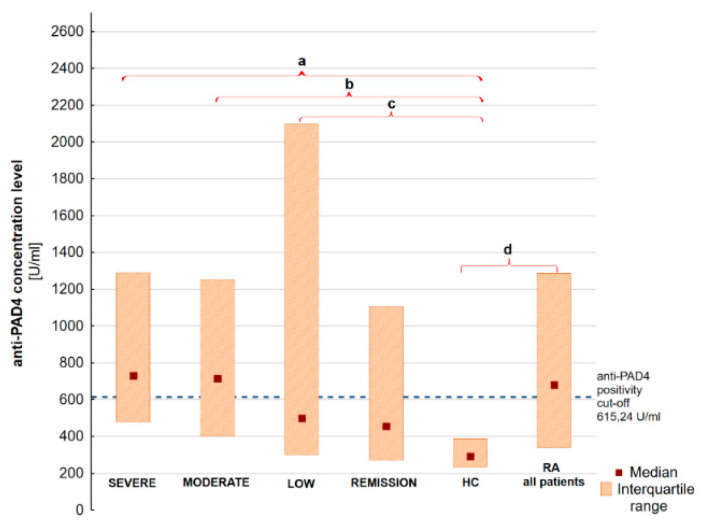
Anti-PAD4 concentration level in RA groups according to DAS28 activity, HC and all RA patients. Results presented as median and interquartile range. (a) *p* = 0.000018, (b) *p* = 0.000095, (c) *p* = 0.038, (d) *p* = 0.00001. RA moderate vs. Remission *p* = 0.054, RA low vs. HC *p* = 0.07.

**Figure 3:**
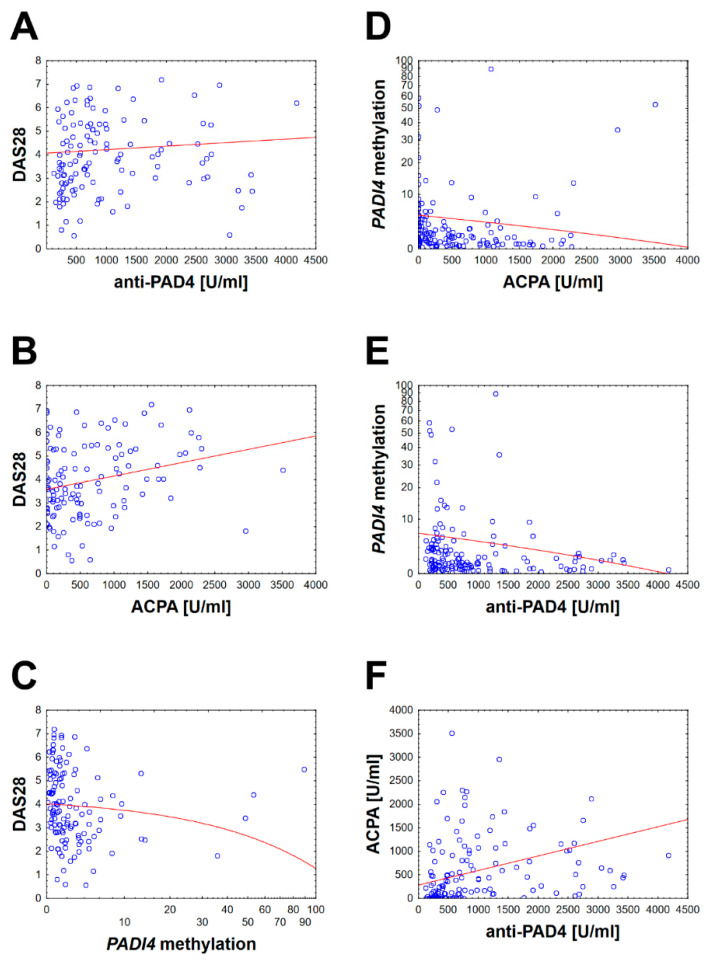
Spearman’s rank correlation between studied parameters. Presented variables are statistically significant with the *p*-value < 0.05. Diagrams from A to F shown correlations between two variables and the following correlation coefficients (r_s_): (**A**) correlation between DAS28 and anti-PAD4 with r_s_ = 0.19; (**B**) correlation between DAS28 and ACPA with r_s_ = 0.22; (**C**) correlation between DAS28 and *PADI4* methylation with r_s_ = −0.34; (**D**) correlation between *PADI4* methylation and ACPA with r_s_ = −0.26; (**E**) correlation between *PADI4* methylation and anti-PAD4 with r_s_ = −0.25; (**F**) correlation between ACPA and anti-PAD4 with r_s_ = 0.52.

We have found that there are significant differences in the anti-PAD4 level between the RA severe and HC group, RA moderate and HC and RA low activity and HC. Moreover, significant differences were discovered in *PADI4* promoter gene methylation between RA severe and RA remission, RA moderate and RA remission and RA moderate and HC. The results are graphically presented in Figure 1 and Figure 2. Numerous correlations of modest or weak strength were also observed between anti-PAD4, *PADI4* gene methylation, ACPA and DAS28. The anti-PAD4 level is associated with DAS28 score. A weak negative correlation between the degree of PAD4 gene promoter methylation and anti-PAD4 aPAD4 concentration, ACPA and the RA disease activity expressed by DAS28 has been found. The correlation is shown in Figure 3. The complete raw results are presented in Supplementary File S1. We have also conducted a multivariate modeling. No factor affecting *PADI4* methylation more strongly than DAS28 was found—see Supplementary File S6.

The additional graphical presentations of our results are showed in Supplementary File S7. No differences in the degree of methylation and anti-PAD4 serum level were found between the groups of patients divided according to the treatment used, including the drugs mechanism of action. (Data available as Supplementary File S3). The characteristics of the study group in subgroups depending on the DAS28 index were collected in Supplementary File S4. No differences other than DAS28 components (ESR, CRP, VAS PGA, VAS PhGA) were found.

## 4. Discussion

Gene expression is subject to overlapping mechanisms of epigenetic regulation, such as methylation of gene promoters, and modifications of histone proteins (methylation, acetylation, phosphorylation, ubiquitination, etc.) Approximately 75% of the genes encoding proteins are regulated through the mechanism of methylation of their promoter regions which are rich in CpG islands [26]. Genetic studies do not explain sufficiently the pathogenesis of RA. Viatte et al. suggest that interaction between genetic and environmental factors are a very promising and poorly understood new area of research [27]. The literature contains no reports concerning the influence of *PADI4* promoter region methylation on the development and course of RA. The results of our research indirectly prove that such an impact exists. The study did not evaluate the concentration or enzymatic activity of PAD4 but the effects of its presence—anti-PAD4 and ACPA. Our results hopefully open this area up for further studies.

The results and trends observed in our study seem to remain in line with the general rule of influence of methylation on gene expression. The reduced *PADI4* methylation in RA patients results in increased expression of PAD enzyme (expression and activity not measured) and, consequently, increased protein citrullination and finally the excessive ACPA production. The results of our study suggest for the first time that the expression of the *PADI4* gene is regulated by the methylation of its promoter region and has an impact on the course of RA. A lower degree of methylation of the *PADI4* gene promoter is associated with the higher activity of RA. The effective treatment leads to a significant increase in methylation of this region, with the highest levels in patients in remission. However, the degree of methylation in effectively treated patients did not reach the level reported in HC. The study results may support the assumption that the higher methylation of CpG islands in the *PADI4* promoter region implies a decrease in the synthesis of the PAD4 enzyme and consequently a reduction in the citrullination of various proteins. We also have found the lower concentration of anti-PAD4 and ACPA, along with a decrease in disease activity.

The subject of our study was not to assess the impact of individual drugs on the level of methylation of the *PADI4* gen, but it is a very interesting issue that requires research. The literature is rich regarding the impact of various drugs for DNA methylation. Differentially methylated positions in DNA have recently been detected in MTX and etanercept treated patients between responders and non-responders. Further research is required to explain their role in RA [28,29].

No single nucleotide polymorphism (SNP) of the *PADI4* gene has been shown to be an important risk factor for RA in the European population [19]. All recognized genetic risk alleles can explain up to 16% of overall disease probability [20]. This indicates the huge importance of environmental factors and their impact on gene expression through epigenetic mechanisms, such as methylation of gene promoters [30]. It has been demonstrated that cigarette smoking, which is one of the environmental factors, stimulates *PADI4* gene expression [21]. The latest research of Meng et al. confirms that gene and smoking-specific interaction may exist, especially in ACPA positive RA patients [31]. The second environmental factor that is suspected to affect the development of RA is the chronic *Porphyromonas gingivalis* infection. It is indicated as the responsible mechanism for the disturbance of citrullination caused by PAD bacterial activity and change in the expression of endogenous PAD [32]. The suggested pathophysiological pathway of RA was shown in Figure 3.

Our research indicates a modest or weak correlation between the low methylation of the *PADI4* promoter region (responsible for high gene expression) and the high activity of the disease. As for RA pathogenesis, it can be concluded that the increased expression of PAD4 through various mechanisms (infection, smoking) results in protein citrullination. Consequently, pre-RA is induced, and then under favorable circumstances, symptomatic RA is developed.

A negative moderate or weak correlation between the methylation of the *PADI4* gene promoter and anti-PAD4, ACPA and DAS28, which was found in our study, may indicate constant modulating effect of citrullination during the course and treatment of RA.

Regardless of the drug used, effective therapy yields a statistically significant increase in methylation of the *PADI4* gene promoter. Because patients from the RA group were treated with various drugs, we can conclude that medicines, regardless of their main mechanism of action, may have an influence on *PADI4* methylation or that the efficacy of treatment may even depend on the final hyper-methylation of this DNA region. The evaluation of DNA methylation seems to be an important source of information useful for the diagnosis, evaluation and treatment of RA. Plant et al. found 5 specific regions that, if hyper-methylated, indicate etanercept non-responder patients [28].

The sensitivity and specificity of anti-PAD4 in the European population of RA patients were estimated at 42% and 92%, respectively. A relationship was also found between the presence of these antibodies and the intensity of radiological changes [9,33]. The prevalence of anti-PAD4 in RA was estimated at 35–45% [34,35]. Our study population of 125 RA patients showed 53.6% of anti-PAD4 positivity for the whole RA group and 7.14% in HC group. The test used in our cohort simultaneously detected IgM, IgA and IgG isotypes of anti-PAD4 antibodies. This may explain the more frequent occurrence of these antibodies in our study. We have observed the decreasing concentration of these antibodies with the lowering disease activity and the smallest concentration in HC (below the cut-off point). The significance of anti-PAD4 in RA is still discussed. Laura Martinez-Prat et al. have analyzed anti-PAD4 in a large cohort of 1473 RA patients and found its discriminative value between RA and HC especially in early RA [36]. Guderud et al. recently showed different results and conclusions that anti-PAD4 is a bystander autoantibody [37]. Taking into account the small number of publication, the usefulness of these antibodies under certain conditions may still be a matter for further evaluation and discussion especially as a prognostic or predictive marker [38].

Our observations regarding the methylation of the *PADI4* promoter prompt us to propose paying more attention to the period when the changes leading to the development of RA start, i.e., before the appearance of inflammatory symptoms. The term pre-RA is now used retrospectively in people who have developed RA; it refers to various stages before the development of any symptoms until the period of unclassified arthritis [39]. Redefinition of pre-RA should be considered in such a way as to stop using the term retrospectively and to use it to make a current diagnosis in specific individuals. This may apply to people who, based on the presence of measurable genetic, serological, epigenetic and other factors are at high risk of developing RA in the future. This creates a need to narrow the current definition of pre-RA only to individuals who have not yet shown any symptoms of inflammation. In the future, this diagnosis may be the basis for the treatment of such patients in the conviction that the symptoms will occur over time. The treatment of such patients will not include anti-inflammatory agents but will be based on affecting epigenetic pathways, for example, by increasing methylation of the *PADI4* promoter in the targeted manner and reducing the synthesis of citrullinated proteins. This may interrupt the pathophysiological pathway and lead to a reduction in ACPA and RF levels and prevent the development of inflammation or prolong the pre-RA phase.

**Figure 4:**
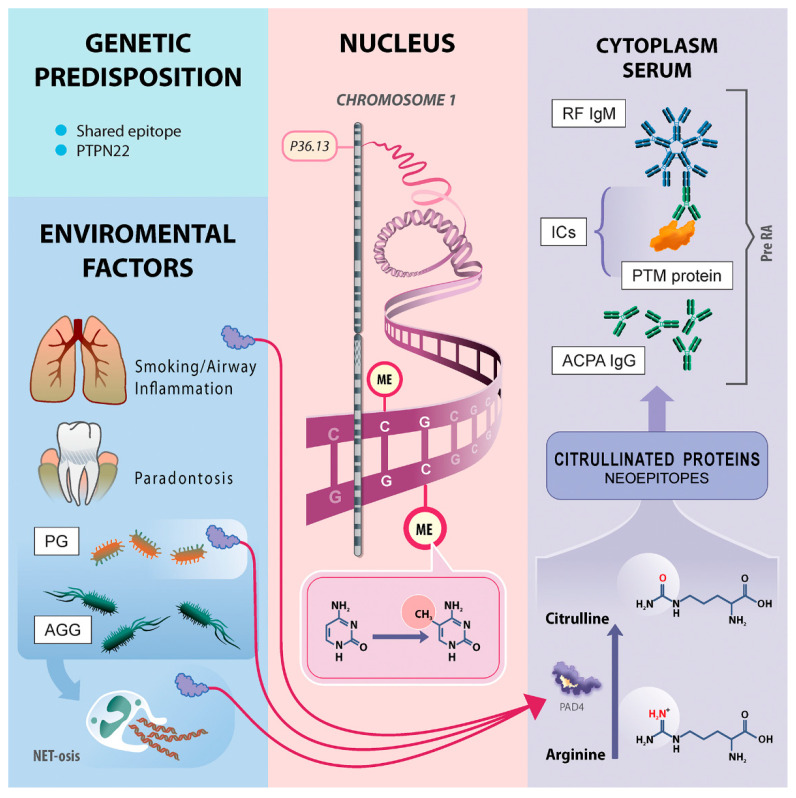
Pathways to RA development. The individuals with genetic predisposition (MHC—shared epitope, SNP of PTPN22) exposed to environmental factors (smoking, airway inflammation, periodontitis) have developed a shift in *PADI4* gene methylation degree. The airway inflammation and smoking are connected with higher PAD activity [40]. NET-osis is a PAD4-dependent mechanism used during the host immunological response against exogenous bacteria like PG and ACC [41,42,43]. Additionally PG is a bacteria with its own PAD4 activity [44]. The dysregulation of PAD4 gene expression via methylation depends on the environmental impact on the mouth and lungs leading to elevated expression of PAD4 enzyme and increased conversion of arginine to citrulline in various proteins, leading to a loss of tolerance to citrullinated proteins and APCA synthesis. The ICs of citrullinated proteins and ACPA IgG become an antigen to RF production. After a period of ACPA and/or RF positivity called pre-RA and unknown “second hit”, the development of arthritis/synovitis is influenced in patients. Abbreviations: PG, *Porphyromonas gingivalis*; AGG, *Aggregatibacter actinomycetemcomitans*; ME, methylation; ICs, immunological complexes; ACPA, anti-citrullinated protein antibodies; PTM, proteins post-translationally modified proteins; PAD4, peptidyl arginine deiminases type 4; RF, Rgeumatoid Factor; Pre RA, pre-Rheumatoid Arthritis; PTPN22, Protein tyrosine phosphatase, non-receptor type 22, NET-osis Neutrophil extracellular traps activation and release.

The most important dependencies of the RA pathogenesis model are gathered in Figure 4. In this model, we postulate that the hypo-methylation of the *PADI4* gene described in our study leads to its excessive expression and increased PAD4 enzyme activity (requires confirmation in further research) and, as a consequence, enhances protein citrullination and breaks immune tolerance leading to synthesis of ACPA. The presented hypothesis may explain the role of hypo-methylation of the *PADI4* gene. However, many questions remain. For example, how environmental factors lead to a decrease in genes methylation.

We realize that the number of 125 patients, especially after dividing according to DAS28 activity score, resulted in difficulties in achieving statistical significance in various comparisons. It is worth indicating the differences in *PADI4* methylation between RA moderate and remission groups (*p*-value = 0.054) and RA low and HC (*p*-value = 0.07). For anti-PAD4 RA remission and HC, we have found a difference with *p* = 0.07. The weak point of our study is that DNA was extracted from the whole blood and not the specific cell line. We did not have the possibility to measure the *PADI4* gene expression, because only frozen whole blood samples were available, and the lack of mRNA expression indicates that our research needs to be supplemented by future studies. Blood samples for testing were obtained in the same way. Patients with infections or severe comorbidities were excluded from the study. However, it should be emphasized that the current study design hinders the elimination of the effect of cellular heterogeneity between studied patients and groups. *PADI4* promoter methylation status might be in unknown degree affected by the diversity of the *PADI4* gene expressing cells. In current literature, there are no studies on methylation of the *PADI4* gene. This is the first research concerning *PADI4* promoter methylation in RA and further investigation is required. The results in specific cell types might be even more distinct and may bring interesting conclusions; similarly, a study concerning histone proteins alterations or interactions between *PADI4* gene and targeted micro-RNAs is needed to better understand the role of *PADI4* gene in RA pathogenesis. It is interesting that in the epigenome studies in the analyzed literature we did not find information indicating the detection of the DMGs in the *PADI4* gene region on chromosome 1.

## 5. Conclusions

In summary, we have presented a novel finding indicating that the *PADI4* gene undergoes epigenetic regulation through methylation of its promoter region. An increase in methylation in this area is associated with lower levels of anti-PAD4 and ACPA and with lower disease activity. The highest methylation status was found in RA remission subgroup and does not depend on the way of treatment. We believe that the expression of PAD4 enzyme might be a key site in RA etiopathogenesis and course. The evaluation of *PADI4* gene promoter methylation can provide a significant value in the early diagnosis of RA, and the DNA region discussed can become a target for RA therapy and even pre-RA treatment in the future. What should be emphasized is that the hypomethylation of the *PADI4* promoter in our study was evaluated in the whole blood cell population, and DNA methylation may differ significantly depending on the assessed cells sub-population and also depending on differences in proportions of synovial fluid or blood cells.

## Figures and Tables

**Table 1:** The characteristic of the rheumatoid arthritis (RA) group, medications and healthy control (HC) group. Anti-PAD4, antibodies against Peptydyl Arginine Deiminase type 4; ACPA, anti-citrullinated protein antibodies; VAS PGA, Visual Analogue Scale Patient Global Assessment; VAS PhGA, Visual Analogue Scale Physician Global Assessment.

Characteristics	RA	HC
	*N* = 125	*N* = 30
Age; mean (SD)	52.2 (12.3)	53.2 (8.1)
Females; *n* (%)	103 (82.4)	23 (76.7)
Disease duration [years]; *n* (SD)	11.69 (9.3)	*n*/*a*
Rheumatoid Factor positive; *n* (%),	87 (69.6)	none
anti-PAD4 positive; *n* (%)	67 (53.6)	2 (6.7)
ACPA positive; *n* (%)	104 (83.2)	none
ESR; mean (SD)	31.5 (24.4)	14.6 (9.2)
CRP [mg/dL]; mean (SD)	13.96 (25.28)	1.37 (1.71)
VAS PGA; mean (SD)	27.1 (27.2)	*n*/*a*
VAS PhGA; mean (SD)	21.7 (20.6)	*n*/*a*
**Treatment**		*n*/*a*
At least Methotrexate, *n* (%)	*90 (72)*	
At least Biologics, *n* (%)	*45 (35.2)*	
At least Steroids, *n* (%)	*73 (58.4)*	
**Single drug therapy, *n* (%)**	45 (36)	
Metotrexate, *n* (%)	23 (18.4)	
Biologics, *n* (%)	5 (4)	
Steroids, *n* (%)	17 (13.6)	
**Double drug therapy, *n* (%)**	50 (40)	
At least Methotrexate + Steroids, *n* (%)	51 (40.8)	
At least Methotrexate + Biologics, *n* (%)	36 (28.8)	
At least Steroids + Biologics, *n* (%)	25 (20)	
Methotrexate + Steroids, *n* (%)	31 (24.8)	
Methotrexate + Biologics, *n* (%)	15 (12)	
Steroids + Biologics, *n* (%)	4 (3.2)	
**Triple drug therapy, *n* (%)**	21 (16.8)	
**No therapy, *n* (%)**	9 (7.2)	

**Table 2:** The *PADI4* promoter methylation degree (methylated sequences) and anti-PAD concentration level in RA group divided according to DAS28, RA patients generally and in healthy control.

	RA Severe DAS28 > 5.1 (*n* = 34; 27.2%)	RA Moderate DAS28 > 3.2–5.1 (*n* = 46; 36.8%)	RA Low DAS28 > 2.6–3.2 (*n* = 19; 15.2%)	RA Remission DAS28 ≤ 2.6 (*n* = 26; 20.8%)	RA Overall *n* = 125	Healthy Control *n* = 30
*PADI4* Metylation [fold change] (methylated sequences) *	1.19 [0.59–1.9]	1.06 [0.54–2.96]	1.66 [1.01–3.22]	2.53 [1.47–4.45]	1.46 [0.68–3.07]	5.17 [2.77–8.74]
anti-PAD4 [U/mL] *	731.03 [477.98–1288.1]	716.11 [400.85–1250.8]	589.57 [306.08–2380.2]	455.54 [271.26–1104.9]	681.39 [339.36–1288.1]	293.25 [234.39–389.12]

* Data are given by median [interquartile range].

## Supplementary Materials

The following are available online at https://www.mdpi.com/2077-0383/9/7/2049/s1, File S1: Raw data PADI4 methylation and anti-PAD4, File S2. Basic data on DAS28, File S3. PADI4 methylation and anti-PAD4 level and RA treatment, File S4. RA group characteristics depending of RA index, File S5. *PADI4* promoter sequence, File S6. Multiple regression analysis, File S7. Graphical presentation of *PADI4* methylation and anti-PAD4 serum level.
