# Supplementary material for: Peptidyl Arginine Deiminase Type 4 Gene Promoter Hypo-Methylation in Rheumatoid Arthritis
Source: J Clin Med. 2020 Jun 30;9(7):2049. doi: 10.3390/jcm9072049 (PMC7408948; doi:10.3390/jcm9072049)
Supplement: Supplementary file 1 [file jcm-09-02049-s001.zip › Supplementary Files 1-7/Supplementary file 2 DAS28.docx]

Supplementary data S2

DAS28

The DAS28 index combines information relating to the number of swollen and tender joints, patient global assessment (measures in 100mm visual analogue scale scale) and erythrocyte sedimentation rate (ESR). The ESR based DAS28 has been well validated for its use in clinical trials.

DAS28 is calculated as follows:

**DAS28 (ESR) = 0.56*√(TJC28)+0.28*√(SJC28)+0.014*PGA+0.70*ln(ESR), where** TJC = tender joint count and SJC = swollen joint count, PGA= patient general assessment

The 28 tender joint count (28TJC) and

28 swollen joint count (28SJC) both range from 0 to 28.

ESR - range from 0 to 150.

PGA - range from 0 to 100.

DAS28 score range from 0 to 9.4.

**Interpretation of scores.**

DAS28n scores table

|  | Remission | Low | Moderate | High |
| --- | --- | --- | --- | --- |
| DAS28 value | **< 2,6** | **2,6-3,2** | **> 3,2-5,1** | **> 5,1** |
