# Supplementary material for: Peptidyl Arginine Deiminase Type 4 Gene Promoter Hypo-Methylation in Rheumatoid Arthritis
Source: J Clin Med. 2020 Jun 30;9(7):2049. doi: 10.3390/jcm9072049 (PMC7408948; doi:10.3390/jcm9072049)
Supplement: Supplementary file 1 [file jcm-09-02049-s001.zip › Supplementary Files 1-7/Supplementary file 3 PADI4 methylation and the treatment methods.docx]

**The relationship between treatment and PADI4 methylation or DAS28 index.**

*Table S2. PADI4 methylation in comparison to the treatment.*

| **Group** | ***PADI4* methylation**  **[methylated sequences]** | | **DAS28** |
| --- | --- | --- | --- |
|  | Median fold-change | Interquartile range | Mean±SD |
| **MTX, n=22 (21%)** | 1.37 | [0.71-3.35] | 4.44±1.65 |
| **MTX+Steroids, n=30 (28.6%)** | 1.17 | [0.65-2.05] | **4.75±1.68** |
| **MTX+Biologics, n=15 (14.3%)** | 2.33 | [1.23-5.25] | **3.12±1.31** |
| **Steroids, n=17 (16.2%)** | 1.49 | [0.48-3.01] | 3.79±1.28 |
| **Triple drugs therapy, n=21 (20)** | 1.68 | [0.87-2.78] | 3.61±1.18 |
| **p-value between groups** | 0.34 | | **0.0037*** |
| **Overall**  **n=105** | 1.43 | [0.69-3.01] | 4.07±1.56 |

*Abbreviations: MTX, methotrexate; SD, standard deviation.*

***** The difference were observed only between groups: MTX+Steroids vs MTX+Biolgics (p=0.006).

*Figure 1. PADI4 methylation in comparison to the treatment.*


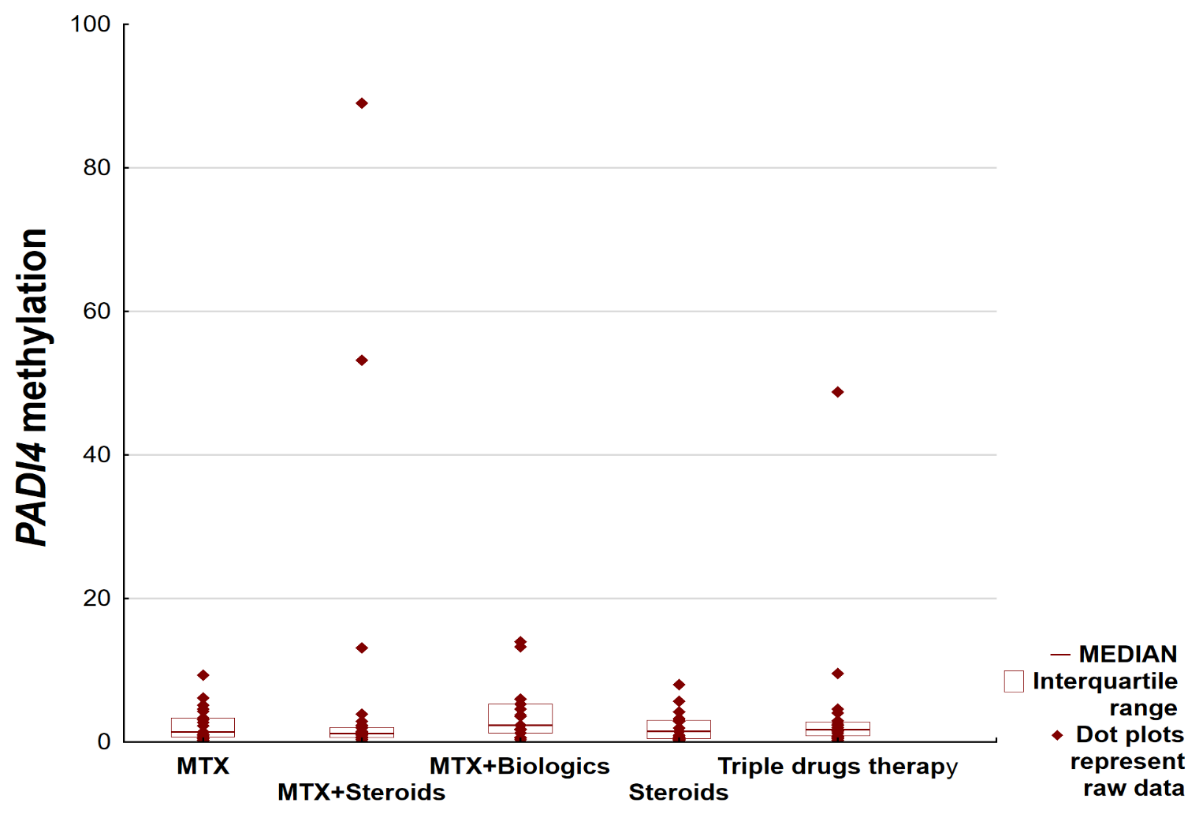


*Abbreviations: MTX, methotrexate*
