# Supplementary material for: Peptidyl Arginine Deiminase Type 4 Gene Promoter Hypo-Methylation in Rheumatoid Arthritis
Source: J Clin Med. 2020 Jun 30;9(7):2049. doi: 10.3390/jcm9072049 (PMC7408948; doi:10.3390/jcm9072049)
Supplement: Supplementary file 1 [file jcm-09-02049-s001.zip › Supplementary Files 1-7/Supplementary file 4 RA group characteristics depending of RA index.docx]

**The characteristics of the RA group depending of RA index**

*Table: The characteristics of the disease activity rheumatoid arthritis groups.*

| **Group** | **Age;** mean (SD) | **Females;** n (%) | **Disease duration** [years]; mean (SD) | **Rheumatoid Factor positive;**  n (%) | **aPAD4 positive;**  n (%) | **ESR;**  mean (SD) | **CRP [mg/dl];** mean (SD) | **VAS PGA;** mean (SD) | **VAS PhGA;** mean (SD) |
| --- | --- | --- | --- | --- | --- | --- | --- | --- | --- |
| **RA Severe**  DAS28˃5.1  N=34; 27.2% | 52 (13.45) | 29 (76.3) | 10.2 (8.8) | 26 (68.4) | 22 (57.9) | 51.3 (25.2) | 31.2 (42.1) | 66 (14.1) | 57.8 (16.5) |
| **RA Moderate**  DAS28˃3.2-5.1  N=46; 36.8% | 53.1 (11.5) | 37 (80.4) | 11.8 (9.9) | 33 (71.7) | 28 (69.9) | 29.6 (20.1) | 11.2 (10.4) | 32.9 (20.3) | 28.1 (17.5) |
| **RA Low**  DAS28˃2.6-3.2  N=19; 15.2% | 55.2 (10.2) | 12  (80) | 14.7 (10.4) | 12 (80) | 7 (46.7) | 31.8 (19.1) | 6.3 (7.2) | 11 (6.3) | 7.8 (5.1) |
| **RA Remission**  DAS28˂2.6  N=26; 20.8% | 48.8 (13.3) | 25 (96.2) | 10.6 (7.3) | 16 (61.5) | 10 (38.5) | 8.6 (7.3) | 2.5 (3.8) | 8.9 (7.2) | 7 (6.5) |
| **p-value between groups** | 0.31 | n/a | 0.41 | n/a | n/a | **<0.0001** | **<0.0001** | **<0.0001** | **<0.0001** |

^*1^ The differences were observed between the following groups: RA Severe vs RA Moderate (p=0.006); RA Severe vs RA Remission (p<0.0001); RA Moderate vs RA Remission (p<0.0001) and RA Low vs RA Remission (p<0.0001).

^*2^  The differences were observed between the following groups: RA Severe vs RA Moderate (p=0.04); RA Severe vs RA Low (p=0.002); RA Severe vs RA Remission (p<0.0001) and RA Moderate vs RA Remission (p=o.0002).

^*3^ The differences were observed between the following groups: RA Severe vs RA RA Moderate (p<0.0001); RA Severe vs RA Low (p<0.0001); RA Severe vs RA Remission (p<0.0001); RA Moderate vs RA RA Low (p-0.003); RA Moderate vs RA Remission (p<0.0001).

^*4^ The differences were observed between the following groups: RA Severe vs RA Moderate (p=0.0002); RA Severe vs RA Low (p<0.0001); RA Severe vs RA Remission (p<0.0001); RA Moderate vs RA Low (p=0.0009) and RA Moderate vs RA Remission (p<0.0001).
