# Supplementary material for: Peptidyl Arginine Deiminase Type 4 Gene Promoter Hypo-Methylation in Rheumatoid Arthritis
Source: J Clin Med. 2020 Jun 30;9(7):2049. doi: 10.3390/jcm9072049 (PMC7408948; doi:10.3390/jcm9072049)
Supplement: Supplementary file 1 [file jcm-09-02049-s001.zip › Supplementary Files 1-7/Supplementary file 5 PADI4 promoter sequence.docx]

***PADI4* promoter sequence.**

In the wide epigenome studies there was no information about methylation sensitive sites in *PADI4* gene [1, 2], thus according to the current methylation protocols [3] the region from Eukaryotic Promoter Database [4] from -1500 to +500 bp relative to transcription start site was chosen for CpG methylation study. MethPrimer software [5] was used for primers design with the following parameters: primer melting temperature between 58-62°C, PCR product length between 80-150 bp and at least 1 CpG must be localized between 1-3 base at the 3’ end of primer to minimalize the specificity. MethPrimer software generate the following results:

*Figure S2. Primers location in the PADI4 gene promoter region.*


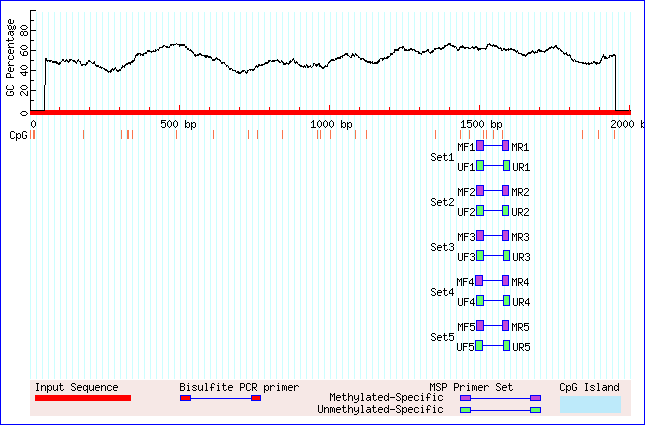


*CpG sites in input sequence were indicated by vertical orange lines.*

All primers pairs were situated in the same region. Please note that one of CpG in figure above in designed PCR product was hidden due to low resolution of generated graphic by software. The detailed primers location (indicated by yellow) was presented below:

1441 AGGTGCTGACTCATGGCCTCTGCTGGGCGATATAAAGGAACCAGCCCAGGGGCTTCCTAC

|||||:|||:|:||||::|:||:||||++|||||||||||::||:::|||||:||::||:

1441 AGGTGTTGATTTATGGTTTTTGTTGGGCGATATAAAGGAATT**AGTTTAGGGGTTTTTTAT**

MF>>>>>>>>>>>>>>>>>

1501 AGCCAGAGGGACGAGCTAGCCCGACGATGGCCCAGGGGACATTGATCCGTGTGACCCCAG

||::|||||||++||:|||::++|++||||:::||||||:||||||:++|||||::::||

1501 **AGTTAGAGGGAC**GAGTTAGTTCGACGATGGTTTAGGGGATATTGATTCGTGTGATTTTAG

>>>>>>>>>>>>

1561 AGCAGCCCACCCATGCCGTGTGTGTGCTGGGCACCTTGACTCAGCTTGACATCTGCAGGT

||:||:::|:::|||:++||||||||:||||:|::||||:|:||:||||:||:||:||||

1561 AGTAGTTTATTTATGT**CGTGTGTGTGTTGGGTATTTTGAT**TTAGTTTGATATTTGTAGGT

MR<<<<<<<<<<<<<<<<<<<<<<<<

***PADI4* promoter sequence from eukaryotic promoter database (EPD):**

>FP000306 PADI4_1 :+U EU:NC; range -1500 to 500.
ATGCGTCAGAACGTCGGACCTGGCCACATCATCATCTGCTGGTACAGAAGATAAATTTTG
AGCCCAGAGAAGGTGGTCTGAATCCAGATCCCCACTGTAGGGCTTATGAGACCTTGGGGG
TCTCACTCAATCTCTCTAAGTCTTTGTGGTCTCATCTACACATGGGCATCCTGATAGGAC
GCACTCCATAGGGATGTTGAGATGAGGCAGGATTTGAACTAAAGCCTGTTTGATCCCACA
GCCTGACTCTGAATTAATGAGGCAAATTCTTTGCTTCAAAGCCAAAGCAAGCAAACAAAA
AACAAACGCAAGATATCCAACCCACGAAACGCTCTATGTTCACGCCAGACTCCCATGCAT
GCACCCTTTGAGTGCTCCTTGTTGGGACCCATCCACCACCCCCCAGTTGCCTCATTAAAG
GCAGAGCCTGGCACCAATGGCCCAGGTGCAACCACAGCTCTGAGGCCACATGGGCATCCC
CCTGGCAGGCGTGGCCCACACCTGCACTGTCTGGTCTGACACCCAGAGGCCCTGGCAAGA
GGCAGGTATCCTGGAGCATGCAGAGAACATCAACTTCCATGCCAGGAAGCTCATCTTCTC
TTCCCAGCTCTGCCGCATTCTAGCTCTGGCACCTTGGGCAAGAGCTAGTCACTTTTACTC
TCTGAGCCTCAATTTTCCTTCTGTAAAATGGGTTGTAATAATAAGCCCATATACACCTTG
TATTGTAGCGAGATGTAGCTGTGATACAGTCTGGAAAGCACGTGTCATATAGACAGGCAG
CTAATGAATCTCACACTCTGGAACTGGACACCTTTGGTTCCAATTCTGGCCCTACCACTC
TCGAGCTGTGGAAGAACTGCACCTCTGTTTTCTCCTCTGTAAAATGGGTATAATAATGGC
ACCAACCTCAGAGGGCTGCTGTGAGGGTGAAATAAGGGGATAATGGTATGATCTAGTTCA
CGGGTTTGTCGTAATGAGCTATTCTATGTGAAGTGCAGGAAAACGTGCCTGGCATAGAGA
ATGTGAGCTGTCATGTGGGTGGCCCAGGTGGAGAGACTTGTCCCAAGCCTGGTTGGTCTT
TGAACGTGTAGCCTGGCCTGTGACTGCTTACCTAAAATCTCCCCGCTTTTCCCACCTTCT
CCTCTCTGATATGCCTGTTTGACATAGGTGAGATTGGATAGATCAAGGTGTTCAGGGCCT
CTAGGCAGGGATGGGACTGTGGGCATGAGGACCAGGACCCAACCCCTCAGCCCCACTCTC
CACCCCAGGCTTTCTGAGCCATCCATCCTTCCCAAGAAACTGACAGAGCCACCCTGCCAC
TGGTACCAGCATTGACACCCATCTAGAGGTCCGAGGGGCAGCCCCAGGGCAGAGGAGATT
TTGAGAGCCCACACCCCTGACCTGAGTGGGGAGGGGTTGAGCCTCTGGGCCACAGACCGC
AGGTGCTGACTCATGGCCTCTGCTGGGCGATATAAAGGAACCAGCCCAGGGGCTTCCTAC
AGCCAGAGGGACGAGCTAGCCCGACGATGGCCCAGGGGACATTGATCCGTGTGACCCCAG
AGCAGCCCACCCATGCCGTGTGTGTGCTGGGCACCTTGACTCAGCTTGACATCTGCAGGT
AAGAGGGGGGCCTTCTGGGGTTTTGGAGGCAGGTCAGGAGATGCTGGATGACCCAGTTCT
ACTGACACAGGAGCATGTGTTTGGCCCAGGCTCTAGGCTCCAGCCTCTGCAGCCACTGCC
AGGGGAGTAGCTGGAGAGAGAAGACCCCAGCAGCCCTGGGAAGTGCCAGTCTCATGGCTG
TGGGTTCTTTGCCATGTACAAGTGCAAATTCCCCAGGAACACACGACAGATAAATTATTA
TGGGGCAAACCAGGCCATCCATCTCCCTGTCTTCATCGAGGCAACAGAAGCTTACAGAGA
GTCTGTGACTTGCTCAAGGTCACACAGCACGTGTGCCCTGGAAGTAAGGTTAGCCCCTGG
GCCTCCTGGTTCACAGCACAG

**SUPPLEMENTARY REFERENCES:**

**1** Liu Y, Aryee MJ, Padyukov L, et al. Epigenome-wide association data implicate DNA methylation as an intermediary of genetic risk in rheumatoid arthritis. *NatBiotechnol.* 2013 ; 31: 142-147.

**2** Shao X, Hudson M, Colmegna I, et al. Rheumatoid arthritis-relevant DNA methylation changes identified in ACPA-positive asymptomatic individuals using methylome capture sequencing. *Clin Epigenetics*. 2019;11(1):110.

**3** Davidović, R.S.; Božović, A.M.; Mandušić, V.L.; Krajnović, M.M. Methylation-specific PCR: four steps in primer design. *Cent. Eur. J. Biol.* 2014, *9*, 1127-1139.

**4** Dreos, R.; Ambrosini, G.; Périer, R.C.; Bucher, P. The Eukaryotic Promoter Database: expansion of EPDnew and new promoter analysis tools. *Nucleic Acids Res.* **2014**, *43*, D92-D96.

**5** Li LC, Dahiya R. MethPrimer: designing primers for methylation PCRs. *Bioinformatics.* 2002;18:1427-1431.
