# Supplementary material for: Peptidyl Arginine Deiminase Type 4 Gene Promoter Hypo-Methylation in Rheumatoid Arthritis
Source: J Clin Med. 2020 Jun 30;9(7):2049. doi: 10.3390/jcm9072049 (PMC7408948; doi:10.3390/jcm9072049)
Supplement: Supplementary file 1 [file jcm-09-02049-s001.zip › Supplementary Files 1-7/Supplementary file 6 Multiple regression analysis.docx]

**Multiple regression analysis between PADI4 methylation and clinical data**

*Table S2. Multiple regression analysis between clinical effects at PADI4 methylation status.*

| **Clinical variable** | **p-value** |
| --- | --- |
| Age | 0.07 |
| Sex | 0.15 |
| Disease duration | 0.78 |
| ESR | 0.74 |
| CRP | 0.36 |
| number of painful joints | 0.75 |
| number of swollen joints | 0.98 |
| VAS PGA | 0.67 |
| VAS PhGA | 0.88 |
| DAS28 | 0.89 |

There was no significant effect of clinical variables to the methylation status in PADI4 gene. For details please refer to Table S2.
