# Supplementary material for: Peptidyl Arginine Deiminase Type 4 Gene Promoter Hypo-Methylation in Rheumatoid Arthritis
Source: J Clin Med. 2020 Jun 30;9(7):2049. doi: 10.3390/jcm9072049 (PMC7408948; doi:10.3390/jcm9072049)

**Dot-plot presentation of *PADI4* methylation and anti-PAD4 serum levels.**

*Figure S1. Dot-plot presentation of PADI4 gene methylation.*


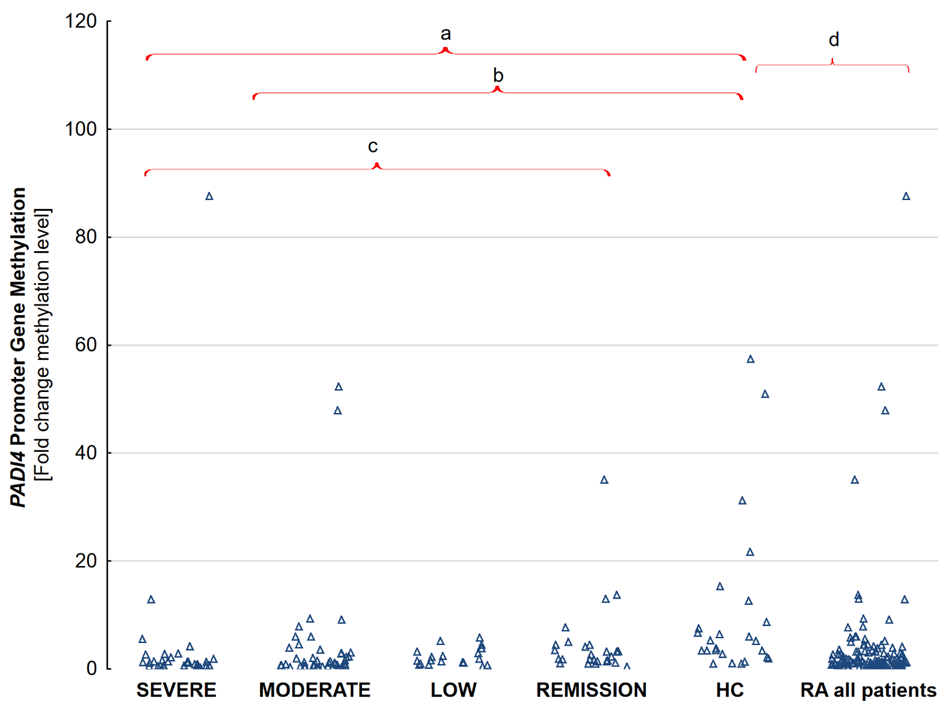


*Figure S2. Dot-plots presentation of anti-PAD4 antibodies serum levels.*


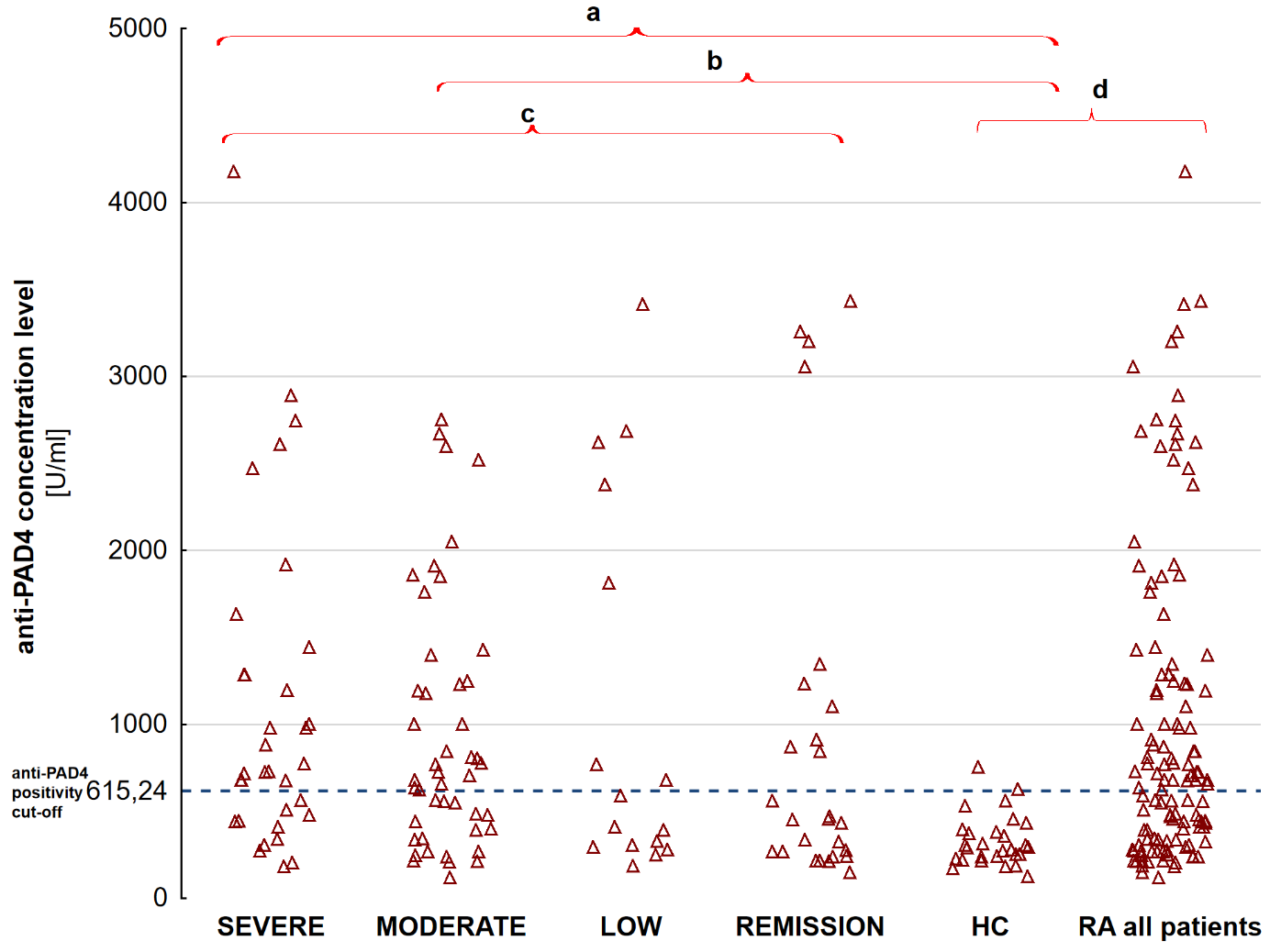

Supplement: Supplementary file 1 [file jcm-09-02049-s001.zip › Supplementary Files 1-7/Supplementary file 7 graphical presentation of PADI4 methylation and anti-PAD4 concentration.docx]
